# Role of Social Support in the Relationship Between Financial Strain and Frequency of Exercise Among Older Japanese: A 19-year Longitudinal Study

**DOI:** 10.2188/jea.JE20190248

**Published:** 2021-04-05

**Authors:** Yukitsugu Komazawa, Hiroshi Murayama, Noboru Harata, Kiyoshi Takami, Giancarlos Troncoso Parady

**Affiliations:** 1 Graduate School of Engineering, The University of Tokyo, Tokyo, Japan; 2 Institute of Gerontology, The University of Tokyo, Tokyo, Japan

**Keywords:** financial strain, social support, exercise, panel data analysis, old age

## Abstract

**Background:**

Previous studies have reported that financial strain has deleterious effects on healthy behaviors. Moreover, social support is expected to mitigate these effects, but few studies have investigated the effects of exercise; thus, the investigation can deepen our understanding of the relationship between social support and physical activity/exercise. We examined the relationship between financial strain and frequency of exercise, and the role of social support in this relationship in old age.

**Methods:**

Data came from a 19-year longitudinal study conducted between 1987 and 2006 of Japanese adults aged 60 or more with up to seven repeated observations. Frequency of exercise was assessed using a four-point scale. Financial strain was measured using the responses to three questions related to financial condition. This study considered both emotional and instrumental supports. Covariates included demographic and socioeconomic factors, health behaviors, and health condition.

**Results:**

The analysis included 3,911 participants. The results of a generalized estimation equation model showed that among females, greater financial strain in the previous wave was associated with reduced frequency of exercise (b = −0.018; 95% confidence interval, −0.032 to −0.004), and that as financial strain increased, those who received more instrumental support engaged in less exercise than those who received less support (b = −0.009; 95% confidence interval, −0.017 to −0.002). These relationships were not observed among males.

**Conclusion:**

This study provides evidence that financial strain is negatively correlated with frequency of exercise among older females. In addition, instrumental support is negatively correlated with frequency of exercise among females under financial strain.

## INTRODUCTION

The World Health Organization stresses the importance of physical activity (PA), and its impact on health outcomes in old age has been widely documented.^1^ PA has been associated with reduced risks of mortality, morbidity, disability, and depression in old age.^2^^–^^5^

In general, exercise is a subset of PA that is planned, structured, and repetitive, and has a specific purpose. One factor affecting the level of PA/exercise is financial strain.^6^^–^^8^ One possible explanation for the association between financial strain and PA/exercise is the cost of participating in an exercise program and/or access to suitable facilities.^9^^–^^11^ This can lead to a negative association (ie, those with higher financial strain tend to engage in less PA/exercise). Another explanation is the stress caused by financial strain. However, stress can be correlated with PA/exercise in both positive and negative ways. For example, studies focusing on older adults have suggested that perceived stress is negatively associated with participation in PA.^12^^–^^17^ Conversely, perceived stress can predict more PA because PA can be used as a coping behavior.^18^ Hence, the relationship between financial strain and PA/exercise can be theoretically bidirectional.

However, to our knowledge, research regarding the relationship between financial strain and PA/exercise remains sparse. Most studies examining the relationship between financial strain and PA/exercise have been based in Western countries.^6^^,^^7^^,^^9^^–^^11^ In contrast, there have been few studies set in non-Western countries. One study of older Japanese adults based on the survey that commenced in 1987 found no association between financial strain and frequency of exercise.^8^ This research needs to be updated because it is more than 30 years old. Moreover, the financial conditions of older Japanese people have changed. A relatively recent report noted that approximately 57% of older Japanese are concerned about financial strain in later life, and this percentage has steadily increased over recent decades.^19^ The proportion of older people who indicated that they lived without financial concerns was 74.7% in 1995 but only 64.6% in 2016. In addition, the financial situation of older people faces downside risk because of longer life expectancy, pension reforms, increased medical expenditure, and aging of non-regular employees.^20^

When considering the relationship between financial strain and PA/exercise, an examination of the mitigating effects of social support on this relationship should provide useful insights and implications. The stress-buffering effects of social support (defined as the protection social support provides from the deleterious effects of stressors like financial strain) on other health-related behaviors, like smoking and alcohol intake, have been investigated.^21^^–^^23^ For instance, those under financial strain who received more general instrumental support were more likely to quit smoking than those who received less support.^23^ However, the mitigating effects of social support on the relationship between financial strain and PA/exercise have not been well investigated. Most studies focusing on the association between financial strain and PA/exercise have been based on cross-sectional or short-term longitudinal data, and thus have failed to account for the time sequence of the relationship.

Summarizing the results of previous studies, two gaps remain to be investigated. First, the association between financial strain and PA/exercise among older Japanese people needs to be examined to provide a better understanding of effective health promotion methods in the Japanese context because previous studies investigating the association between financial strain and PA/exercise have mainly been conducted in Western countries.^6^^,^^7^^,^^9^^–^^11^ Second, to the best of our knowledge, the buffering effect of social support on the relationship between financial strain and PA/exercise has not yet been examined.

Thus, to address these gaps, this study investigated the relationship between financial strain and frequency of exercise and the mitigating effects of social support on this relationship using data from a 19-year longitudinal study of older Japanese people. Two hypotheses were examined: older people who are under more financial strain engage in less exercise (H_1_), and social support buffers the deleterious effects of financial strain on frequency of exercise (H_2_).

## METHOD

### Sample and procedures

The samples were taken from the National Survey of the Japanese Elderly (NSJE), which was a seven-wave (1987, 1990, 1993, 1996, 1999, 2002, and 2006) longitudinal survey using equivalent questions in each wave. At the commencement of the survey, 2,200 people aged 60 or more were recruited. However, because of attrition, the original sample was supplemented with new participants in 1990 (*N* = 580) and 1996 (*N* = 1,210), and an additional group of participants aged 70 or older (*N* = 2,000) was added in 1999. Participants were chosen using the random sampling method and data collection was based on face-to-face interviews, with trained interviewers visiting the respondents’ homes and asking a series of questions based on a structured questionnaire. Overall response rates ranged from 67.2% to 92.6% (Wave 1: 2,200 [67.2%], Wave 2: 2,227 [85.2%], Wave 3: 2,061 [92.6%], Wave 4: 2,751 [86.0%], Wave 5: 3,989 [85.2%], Wave 6: 3,245 [83.7%], and Wave 7: 2,459 [75.4%]).^24^

Proxy interviews were conducted for those who could not be interviewed because of health problems. However, because the proxy interviews did not cover the main variables used in this study, they were excluded. As a result, data were obtained from 4,869 individuals aged 60 or more. This study was approved by the Institutional Review Board of the Tokyo Metropolitan Institute of Gerontology.

### Measures

#### Frequency of exercise

In this study, frequency of exercise was used as the explained variable. Frequency of exercise was measured using the question “How often do you do exercise?” A higher score indicated greater frequency of exercise (usually [4], often [3], seldom [2], or never [1]).

#### Financial strain

Financial strain was used as the main explanatory variable based on a three-item composite score relating to (a) satisfaction with finances (“Are you satisfied with your financial condition?”; completely satisfied, very satisfied, neither, not very satisfied, or not at all satisfied), (b) objective financial situation compared with others (“Is your financial condition better than others?”; better, about the same, or worse), and (c) whether they had enough spending money (“Do you have enough pocket money?”; yes or no).

To ensure that the variables were treated equally, the minimum and maximum for each variable were set at the same level. Thus, we scored the binary question (c) as either 1 or 5 points (ie, yes [1] or no [5]) and the three-point question (b) as either 1, 3, or 5 points (ie, better [1], about the same [3], or worse [5]), as the other variable (a) was measured on a five-point scale (ie, completely satisfied [1] to not at all satisfied [5]). Cronbach’s alpha was 0.67. The composite scores ranged from 3 to 15, with higher scores indicating greater financial strain.

#### Social support

In this study, one of the main purposes was to investigate the mitigating effects of social support. Therefore, social support was used as an explanatory variable. In general, many researchers have agreed that there are two types of social support, and based on this perspective, this study divided social support into emotional support and instrumental support. Each of these was measured by a composite score based on two questions with five response categories (never [1] to always [5]). The questions regarding emotional support were “How often does someone listen to you?” and “How often does someone show you love and understanding?” The questions regarding instrumental support were “How often does someone care for you when you are sick?” and “How often does someone provide financial assistance?” Both composite scores ranged from 2 to 10 (Cronbach’s alpha was 0.87 for emotional support and 0.61 for instrumental support), and a higher score indicated more support.

#### Covariates

Demographic and socioeconomic variables, health behaviors (body mass index [BMI], and smoking behavior), and health conditions (functional health, depression symptoms, cognitive impairment, self-rated health, and chronic diseases) were included as covariates. These variables were considered to be the determinants or factors affecting PA/exercise in previous studies.^25^

Baseline age, gender, marital status (married or unmarried), and working status (working or not working) were included as demographic variables. Annual household income and educational level were included as socioeconomic variables. Education level was divided into three categories: ≤9 years, 10–12 years, or ≥13 years. Annual household income was measured using a five-point scale: <1.2 million yen [1], 1.2–2.9 million yen [2], 3.0–4.9 million yen [3], 5.0–9.9 million yen [4], or ≥10 million yen [5]. Household income provides an objective representation of financial status, while financial strain provides a subjective representation. Therefore, we used both household income and financial strain in this study. We checked for multicollinearity by observing the correlation between annual household income and financial strain, and confirmed that there was little evidence of multicollinearity. BMI was calculated based on self-reported weight and height (kg/m^2^).

Functional health was measured using six basic activities of daily living (BADL) items and five instrumental activities of daily living (IADL) items.^26^^,^^27^ Regarding the IADL items, all items except climbing stairs and walking a few blocks were coded from no difficulty [1] to unable to do [5]. These two items were measured using a five-point scale in Waves 1 and 2 and a four-point scale in Waves 3 through 7. To treat the observations from each wave equally, the four-point scale used in Waves 3 through 7 was transformed into a five-point scale by multiplying scores by 5/4. This method was also used in a previous study.^23^ All of the resulting scores were rounded to the nearest integer. As a result, total scores ranged from 6 to 30 for the BADL items and from 6 to 25 for the IADL items (Cronbach’s alpha = 0.92), with higher scores indicating greater functional disability.

Depressive mood was measured using the Center for Epidemiological Studies Depression Scale (CES-D), which resulted in scores ranging from 0 to 27, with higher scores indicating worse mental health.^28^ Cognitive impairment was assessed using Pfeiffer’s Short Portable Mental Status Questionnaire, which is a ten-item screening instrument.^29^ We used nine of Pfeiffer’s items, excluding the question relating to the subject’s telephone number.^30^ Scores ranged from 0 to 9, with higher scores indicating more severe cognitive impairment. Self-rated health was evaluated using three items measured on a scale from 1 to 5, with composite scores ranging from 3 to 15 (Cronbach’s alpha = 0.85). Higher scores indicated poorer self-rated health. Chronic diseases included hypertension, heart disease, diabetes mellitus, stroke, and arthritis.

### Data analysis

Panel data analysis is well-suited to studies of changes over time using repeated measures of the same individual. In this study, panel data analysis was performed using a generalized estimation equation (GEE). To consider the time sequence, lagged variables were used as explanatory variables. The lagged variables were taken from the previous wave (t–1) and the time interval between each wave was three or four years. To minimize the effects of missing variables, three different datasets were imputed using NORM software.^31^ By using the multiple imputation method, we were able to include every participant in the estimation. Based on the missing-at-random assumption, the missing variables were imputed using the Markov chain Monte Carlo method. The parameter estimates were averaged, and their variances were adjusted based on the three imputation datasets.^32^ Here, to avoid multicollinearity, all of the variables were centered to the group mean of the survey wave.

The analysis was based on two models. One used lagged variables without any interaction terms and the other included the interaction terms (instrumental/emotional support and financial strain). In addition, to investigate whether there was a gender difference, the analyses were conducted on both males and females. Statistical significance was set as *P* < 0.05. All of the statistical analyses were performed using Stata/SE software version 14.2 (StataCorp, College Station, TX, USA).

## RESULTS

We included 3,911 samples in the analysis after excluding those who were observed only once during the study period. Baseline descriptive statistics are shown in Table 1. The average age was 68.9 years, 56.6% of subjects were female, and the average period of education was 9.2 years. In terms of frequency of exercise, the proportion of respondents in each category was around 25%.

**Table 1.** Baseline descriptive statistics (*N* = 3,911)

		Mean	SD	%
Exercise		2.5	1.1	
	Usually			26.4
	Often			23.2
	Seldom			27.0
	Never			23.5
Financial strain	(range: 3–15)	8.3	2.2	
Emotional support	(range: 2–10)	8.3	1.7	
Instrumental support	(range: 2–10)	8.1	1.5	
Baseline age		68.9	6.7	
Gender	Female			56.6
Marital status	Married			64.6
Working status	Working			29.4
Years of education		9.2	2.6	
	≤9			60.6
	10–12			29.3
	≥13			10.1
Annual household income		2.9	1.1	
	<1.2 million yen			9.6
	1.2–2.9 million yen			27.6
	3–4.9 million yen			30.7
	5–9.9 million yen			23.6
	≥10 million yen			8.5
BMI, kg/m^2^		22.2	3.2	
Smoking behavior	Smoking			22.2
BADL	(range: 6–30)	6.3	1.6	
IADL	(range: 6–25)	6.3	2.7	
Depression	(range: 0–27)	9.6	2.4	
Cognitive impairment	(range: 0–9)	1.0	1.3	
Self-rated health	(range: 3–15)	7.5	2.8	
Chronic diseases				
Hypertension	Yes			31.1
Heart disease	Yes			13.7
Diabetes mellitus	Yes			8.1
Stroke	Yes			3.6
Arthritis	Yes			18.4

BADL, basic activities of daily living; BMI, body mass index; IADL, instrumental activities of daily living; SD, standard deviation.

The results of the GEE model analysis are shown in Table 2 and Table 3. Table 2 (without interaction terms) shows that among females, financial strain in the previous wave had a statistically significant association with frequency of exercise (b = −0.018; 95% confidence interval (CI), −0.032 to −0.004) after controlling for covariates including frequency of exercise in the previous wave. This indicates that as financial strain increased, older females were less likely to undertake exercise. This association was not found among males.

**Table 2.** Generalized equation estimation model for frequency of exercise (without interaction terms)

	Male			Female		
	
	b	(95% CI)	*P*	b	(95% CI)	*P*
Financial strain, every one point	−0.012	(−0.029, 0.005)	0.172	−0.018	(−0.032, −0.004)	0.011
Emotional support, every one point	0.007	(−0.012, 0.026)	0.482	0.006	(−0.011, 0.022)	0.497
Instrumental support, every one point	−0.011	(−0.033, 0.011)	0.329	0.006	(−0.012, 0.024)	0.521
Baseline age, years	−0.009	(−0.016, −0.002)	0.008	−0.020	(−0.026, −0.014)	<0.001
Married	0.010	(−0.106, 0.126)	0.868	−0.042	(−0.108, 0.023)	0.204
Working	−0.151	(−0.222, −0.081)	<0.001	−0.012	(−0.081, 0.056)	0.726
Years of education^a^						
10–12	0.074	(−0.017, 0.166)	0.110	0.136	(0.062, 0.209)	<0.001
≥13	0.244	(0.140, 0.347)	<0.001	0.320	(0.211, 0.430)	<0.001
Annual household income, every one class	−0.011	(−0.045, 0.023)	0.527	−0.007	(−0.033, 0.019)	0.610
BMI, kg/m^2 b^						
<18.5	−0.147	(−0.259, −0.035)	0.010	−0.033	(−0.122, 0.056)	0.465
≥25.0	−0.011	(−0.105, 0.082)	0.814	−0.100	(−0.170, −0.030)	0.005
Smoking	−0.179	(−0.251, −0.106)	<0.001	−0.067	(−0.187, 0.054)	0.277
BADL, every one point	0.030	(−0.012, 0.073)	0.163	−0.015	(−0.047, 0.016)	0.339
IADL, every one point	−0.084	(−0.113, −0.055)	<0.001	−0.020	(−0.038, −0.002)	0.028
Depression, every one point	0.006	(−0.008, 0.020)	0.412	0.005	(−0.005, 0.016)	0.312
Cognitive impairment, every one point	−0.024	(−0.053, 0.005)	0.104	−0.022	(−0.044, 0.000)	0.054
Self-rated health, every one point	−0.058	(−0.073, −0.044)	<0.001	−0.043	(−0.055, −0.030)	<0.001
Hypertension	−0.026	(−0.100, 0.049)	0.504	−0.047	(−0.107, 0.013)	0.128
Heart disease	−0.018	(−0.121, 0.086)	0.739	−0.038	(−0.120, 0.043)	0.358
Diabetes mellitus	0.030	(−0.088, 0.147)	0.621	0.019	(−0.099, 0.138)	0.748
Stroke	−0.054	(−0.216, 0.109)	0.519	0.175	(0.002, 0.347)	0.047
Arthritis	−0.060	(−0.159, 0.040)	0.242	0.031	(−0.032, 0.095)	0.335
Constant term	3.210	(2.746, 3.674)	<0.001	3.860	(3.475, 4.244)	<0.001

BADL, basic activities of daily living; BMI, body mass index; CI, confidence interval; IADL, instrumental activities of daily living.

^a^Reference category was “≤9 years”.

^b^Reference category was “18.5–24.9”.

**Table 3.** Generalized equation estimation model for frequency of exercise (with interaction terms)

	Male			Female		
	
	b	(95% CI)	*P*	b	(95% CI)	*P*
Financial strain, every one point	−0.011	(−0.027, 0.006)	0.208	−0.014	(−0.027, −0.001)	0.038
Emotional support, every one point	−0.001	(−0.020, 0.017)	0.877	0.006	(−0.010, 0.022)	0.465
Instrumental support, every one point	−0.009	(−0.031, 0.013)	0.431	0.005	(−0.013, 0.023)	0.606
Emotional support x Financial strain	0.000	(−0.008, 0.008)	0.986	0.005	(−0.002, 0.012)	0.194
Instrumental support x Financial strain	−0.004	(−0.014, 0.005)	0.387	−0.009	(−0.017, −0.002)	0.018
Baseline age, years	−0.008	(−0.014, −0.003)	0.004	−0.016	(−0.021, −0.012)	<0.001
Married	−0.011	(−0.112, 0.089)	0.827	−0.040	(−0.095, 0.016)	0.159
Working	−0.128	(−0.194, −0.062)	<0.001	0.002	(−0.061, 0.065)	0.961
Years of education^a^						
10–12	0.058	(−0.016, 0.131)	0.123	0.103	(0.046, 0.160)	<0.001
≥13	0.173	(0.090, 0.256)	<0.001	0.239	(0.153, 0.326)	<0.001
Annual household income, every one class	−0.006	(−0.038, 0.026)	0.713	−0.011	(−0.036, 0.014)	0.377
BMI, kg/m^2 b^						
<18.5	−0.126	(−0.230, −0.022)	0.018	−0.030	(−0.110, 0.050)	0.462
≥25.0	−0.007	(−0.092, 0.079)	0.878	−0.095	(−0.158, −0.033)	0.003
Smoking	−0.153	(−0.217, −0.090)	<0.001	−0.099	(−0.201, 0.003)	0.058
BADL, every one point	0.028	(−0.012, 0.069)	0.171	−0.007	(−0.038, 0.023)	0.635
IADL, every one point	−0.067	(−0.096, −0.039)	<0.001	−0.013	(−0.030, 0.004)	0.135
Depression, every one point	0.002	(−0.012, 0.015)	0.826	0.003	(−0.007, 0.013)	0.598
Cognitive impairment, every one point	−0.018	(−0.047, 0.010)	0.202	−0.017	(−0.039, 0.004)	0.113
Self-rated health, every one point	−0.049	(−0.063, −0.035)	<0.001	−0.030	(−0.042, −0.019)	<0.001
Hypertension	−0.015	(−0.084, 0.053)	0.663	−0.038	(−0.093, 0.016)	0.169
Heart disease	−0.022	(−0.118, 0.074)	0.656	−0.024	(−0.100, 0.051)	0.525
Diabetes mellitus	0.018	(−0.088, 0.124)	0.744	−0.025	(−0.132, 0.082)	0.645
Stroke	−0.041	(−0.195, 0.114)	0.605	0.156	(−0.007, 0.320)	0.061
Arthritis	−0.047	(−0.144, 0.049)	0.337	0.018	(−0.043, 0.079)	0.565
Constant term	3.137	(2.750, 3.523)	<0.001	3.618	(3.306, 3.931)	<0.001

BADL, basic activities of daily living; BMI, body mass index; CI, confidence interval; IADL, instrumental activities of daily living.

^a^Reference category was “≤9 years”.

^b^Reference category was “18.5–24.9”.

Regarding the other economic variable, annual household income in the previous wave was not statistically significant. A higher education level had a statistically significant association with greater frequency of exercise among both males and females, while annual household income did not. Neither instrumental support nor emotional support in the previous wave was associated with frequency of exercise.

**Figure 1.**
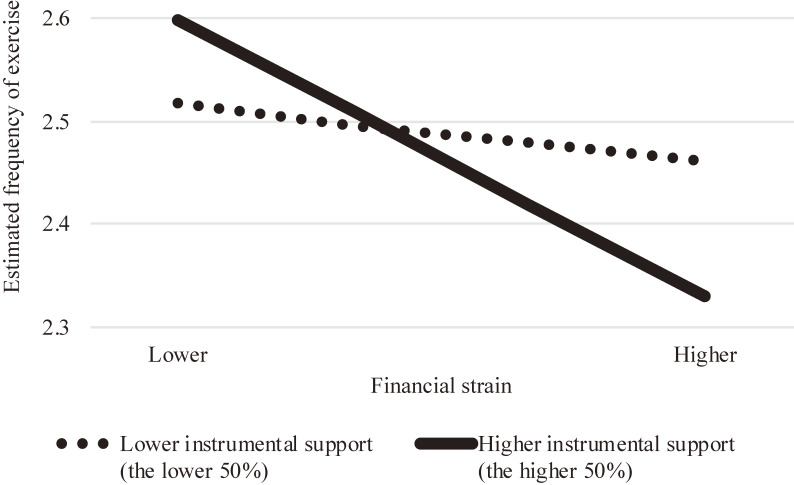
Relationship between financial strain, instrumental support, and frequency of exercise among older females. Estimated frequency of exercise (y-axis) was calculated after adjusting for all of the variables other than financial strain and instrumental support. Scores for frequency of exercise ranged from 1 to 4 (*never* [1], *seldom* [2], *often* [3], or *usually* [4]).

Table 3 (with interaction terms) shows that the interaction between instrumental support and financial strain in the previous wave was statistically significant among females (b = −0.009; 95% CI, −0.017 to −0.002), but not among males. Figure 1 shows frequency of exercise in relation to financial strain and instrumental support. Estimated frequency of exercise (y-axis) was calculated after adjusting for all of the variables other than financial strain and instrumental support. Figure 1 indicates that under higher financial strain, those who had more instrumental support exercised less frequently than those who had less instrumental support. Conversely, the interaction term between emotional support and financial strain was not statistically significant for either gender.

## DISCUSSION

This study investigated the relationship between financial strain and frequency of exercise and the role of social support in mitigating this relationship for older Japanese people. The findings provide two main insights. First, financial strain was negatively associated with frequency of exercise among older Japanese females. Second, older females under financial strain who were receiving more instrumental support were less likely to undertake exercise.

Higher financial strain was associated with less exercise among older females, which partly supports our first hypothesis (H_1_). Regarding the causal nature of the relationship, possible explanations include stress caused by financial strain, constraints such as time and financial resources, barriers to exercise opportunities as a result of limited financial capacity, and the perception that it would be more appropriate to spend whatever time and money they have on other, more necessary things.^33^^,^^34^

In addition, the association was only found among females, suggesting that there might be a gender difference. Because a longitudinal study using a national Japanese sample reported that in 69.5% of households, the household finances were managed by the female partner,^35^ it is possible that females were more likely to feel financial strain. Thus, financial strain possibly had more influence on frequency of exercise among females.

Neither instrumental support nor emotional support was significantly associated with frequency of exercise. It is known that in general, social support specific to PA/exercise has a positive effect on PA/exercise because it facilitates easier access to resources and materials that are useful for PA/exercise, provides advice that encourages people to continue to exercise, and provides social norms.^36^^–^^39^ Because this study examines the role played by general social support (ie, not specifically related to PA/exercise), our findings are not consistent with those of previous studies.

The interaction term between financial strain and instrumental support was statistically significant among females. As can be seen from Figure 1, under higher levels of financial strain, those who received more instrumental support engaged in less exercise than those who received less support. However, the interaction term between emotional support and financial strain was not statistically significant. These findings indicate that our second hypothesis (H_2_) was not supported. There are three possible reasons for the significant interaction term between instrumental support and financial strain; reduced stress because of the stress buffering effects of social support, the stigma caused by receiving support, and residual confounding.

First, if exercise is undertaken with the intention of coping with stress, receiving more social support, which buffers the effects of financial strain, can reduce the frequency of exercise. For example, in the case of smoking behavior, smoking is a coping behavior, and reduced stress can increase the likelihood of quitting smoking behavior.^23^ Second, regarding the stigma associated with receiving support from others, empirical studies have found that when people receive social support, especially instrumental support, their mental health tends to decline.^40^ Further, Japanese people are likely to consider the receipt of instrumental support from others as a sign that they are dependent, and thus as shameful.^41^ If older people view exercise as an unnecessary activity (such as leisure activities), this sense of shame at receiving support might make people in need reluctant to engage in exercise. Third, there might be residual confounding of the associations among financial strain, instrumental support, and frequency of exercise, even though we adjusted for several variables including health conditions. That is, those who are physically, mentally, and socially vulnerable might tend to have a higher probability of experiencing financial strain, require more instrumental support, and engage in less exercise.

Unlike instrumental support, there was no statistically significant interaction between emotional support and financial strain. Previous studies revealed that receiving emotional support is not expected to have a negative impact on people’s sense of independence.^40^ Therefore, emotional support might not have the same moderating effect as instrumental support.

There are several limitations. First, frequency of exercise was self-reported, and a single indicator might be insufficient to comprehensively evaluate the level of PA other than exercise. The use of pedometers would provide a more objective measure of the level of PA and enable more beneficial insights. Second, various policies aimed at promoting healthy lifestyles were initiated in Japan during the period of observation from 1987 to 2006 (eg, ‘Healthy Japan 21’ in 2000), but these environmental changes were not taken into account in this study. Third, the time interval of 3–4 years between waves presents difficulties because the condition of older people could have changed and specific incidents affecting their frequency of exercise (eg, hospitalization) could have occurred during this period. Therefore, the result of this study might be sufficient to explain the association between financial strain and frequency of exercise, but might not be sufficient to explain the magnitude of the effects of financial strain.

In conclusion, this study, which was based on a 19-year longitudinal study of older Japanese people, revealed that higher financial strain was associated with less frequency of exercise among females, but not among males. In addition, regarding the role of social support, greater instrumental support was negatively related to frequency of exercise under financial strain among females. Individuals experience different financial conditions, and thus the investigation of the effects of financial strain on people’s behavior can enhance our understanding of the importance of improving the financial environment surrounding people (eg, the economic climate). Moreover, the role of social support, including its stress-buffering effect, might be influenced by the context, such as people’s individual situations and the community culture in which they live.
